# Integrated expression profiles of mRNA and microRNA in the liver of Fructus Meliae Toosendan water extract injured mice

**DOI:** 10.3389/fphar.2015.00236

**Published:** 2015-10-19

**Authors:** Jie Zheng, Cai Ji, Xiaoyan Lu, Wei Tong, Xiaohui Fan, Yue Gao

**Affiliations:** ^1^Pharmaceutical Informatics Institute, College of Pharmaceutical Sciences, Zhejiang UniversityHangzhou, China; ^2^Department of Pharmacology and Toxicology, Beijing Institute of Radiation MedicineBeijing, China

**Keywords:** DILI, Fructus Meliae Toosendan (FMT), miRNA, mRNA

## Abstract

Liver toxicity is a severe problem associated with Traditional Chinese Medicine (TCM). Fructus Meliae Toosendan (FMT) is a known hepatotoxic TCM, however, the toxicological mechanisms of liver injury caused by FMT treatment still remain largely unknown. In this study, we aimed to reveal possible mechanisms of FMT water extract-induced liver injury using a systemic approach. After three consecutive daily dosing of FMT water extract, significant increases of alanine transaminase, aspartate transaminase, and alkaline phosphatase activities, along with elevated total bilirubin and total cholesterol levels and a decrease of triglyceride level, were detected in mice serum. Moreover, hydropic degeneration was observed in hepatocytes, suggesting the presence of FMT-induced liver injury. mRNA and microRNA expression profiles of liver samples from injured mice were analyzed and revealed 8 miRNAs and 1,723 mRNAs were significantly changed after FMT water extract treatment. For the eight differentially expressed miRNAs, their predicted target genes were collected and a final set of 125 genes and 4 miRNAs (miR-139-5p, miR-199a-5p, miR-2861, and miR-3960) was selected to investigate important processes involved in FMT hepatotoxicity. Our results demonstrated several cellular functions were disordered after FMT treatment, such as cellular growth and proliferation, gene expression and cellular development. We hypothesized that liver cell necrosis was the main liver toxicity of FMT water extract, which was possibly caused by oxidative stress responses.

## Introduction

Drug-induced liver injury (DILI) is the main cause of drugs being removed from the market and it is a universal side effect of drugs (Halegoua-De and Navarro, 2008). Over 80% FDA-approved prescription drugs with the black box warning are related to severe DILI (Chen et al., 2011). Since liver plays a very important role in the detoxification of xenobiotics and it is prone to generate chemically reactive metabolites, it makes the liver being the target organ of DILI (Hornby et al., 2014). The most common histological patterns of DILI in clinic are the acute (21%) and chronic hepatitis (14%), acute (9%), and chronic cholestasis (10%), and cholestatic hepatitis (29%) (Kleiner et al., 2014). Among them, biochemical presentations of hepatocellular injury, cholestatic injury, and both are all detected in these histological patterns (Kleiner et al., 2014). It is worth to mention that the reports of Traditional Chinese Medicine (TCM)-induced liver injury are on the rise from many countries worldwide as it has been used more frequently. Liver injury becomes one of the main toxicities caused by TCM. Due to the characteristics of TCM, e.g., multi-components and multi-targets, it is difficult to understand the mechanism of TCM-induced liver injury.

Microarray provides more sensitive and detailed insights into the mechanisms of toxic action at an earlier molecular level and has been employed as a useful method to study the mechanisms of DILI (Lizarraga et al., 2012). Results from mRNA microarray analysis can also correlate to the observations from histopathology and clinical chemistry (Waring et al., 2001), which could offer further insight into the mechanisms of DILI.

In recent years, microRNAs (miRNAs) have drawn considerable attentions because of their critical effects on cellular processes, pathological processes, and physiological processes (Kloosterman and Plasterk, 2006). miRNAs are short noncoding single-stranded RNAs of approximately 22 nucleotides that decrease the expressions of target mRNAs (Giraldez et al., 2006; Guo et al., 2010) or influence protein production (Wightman et al., 1993; Olsen and Ambros, 1999) by binding to target mRNAs. miRNAs have been demonstrated to play an important role in DILI. For example, miR-122, the most abundant miRNA in liver, interacts with many targets involved in metabolism and stress-response pathways (Bala et al., 2012) and could be a potential biomarker of DILI (Starkey et al., 2011). miRNA microarray is a powerful tool to detect global alteration of miRNA levels and their functions during liver injury. Wang et al (Wang et al., 2009) showed that some specific miRNA species in blood, such as miR-122 and miR-192, have detectable dose- and exposure duration-dependent changes in APAP-induced liver injury in mice using miRNA microarray. In addition, these miRNAs were found to change at an even earlier stage than the traditional chemical markers used in clinic, such as alanine transaminase (ALT).

Integration of mRNA microarray and miRNA microarray would provide a more comprehensive understanding on mechanisms of DILI. This approach has been examined by Lizarraga et al. (2012) to evaluate benzo[α]pyrene (BaP)-induced toxicity at genetic level and eight differentially expressed miRNAs seemed to be involved in the pathways of apoptotic signaling, cell cycle arrest, DNA damage response, and DNA damage repair, thus leading to liver injury.

**FIGURE 1:**
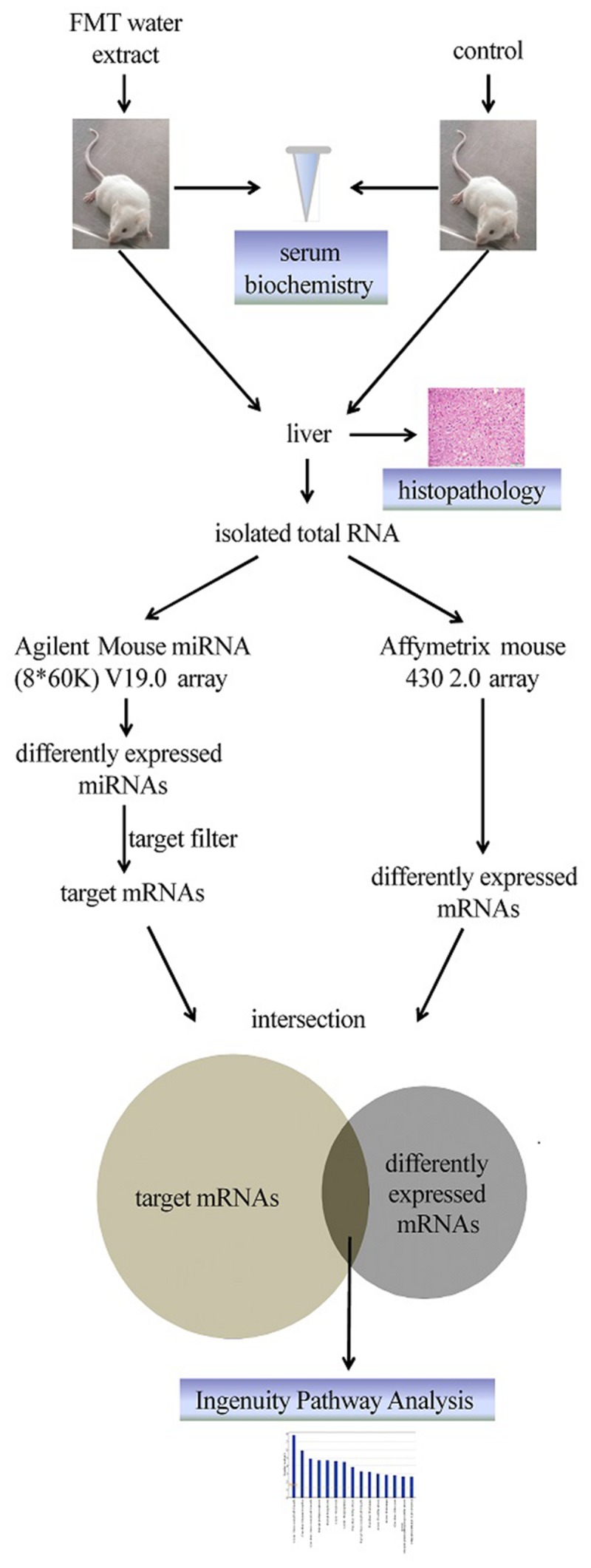
**Scheme for this study**.

In this study, Fructus Meliae Toosendan (FMT) was used as a model drug, which is a typically hepatotoxic TCM. FMT has been used generally as an insecticidal and medicinal plant for a long time in China and Korea to treat stomach ache, cholelithiasis, cholecystitis, gastritis, mastitis, and ascariasis (Xie et al., 2008). Liver injury caused by FMT has been reported frequently in recent years (Yuen et al., 2006). Most attention has been concentrated on the single component of FMT, namely toosendanin, which has been identified as one of the toxic compounds based on the increase in serum glutamic pyruvic transaminase (SGPT) and changes in histopathology. Several toxicity mechanisms of toosendanin have been proposed, such as mitochondrial dysfunction, caspase induction, and ROS and MAP kinases (Zhang et al., 2008). However, it remains a question if toosendanin is solely responsible for FMT-induced liver injury since the clinical application form of FMT comprises of complex components. Moreover, TCM prescriptions are usually water-based solutions as well as FMT. Thus, it is interesting to investigate toxicological mechanisms of FMT water extract-induced liver injury. Here we applied an integrated approach of miRNA and mRNA expression profiles to systemically elucidate the potential toxicological mechanisms of FMT water extract-induced liver injury. **Figure 1** summarized the overall research strategy and experimental design for this study.

## Materials and Methods

### Extraction of FMT

Dried whole FMT (6 kg, purchased from Zhejiang Chinese Medical University Medicine Plant, Hangzhou, China) was infused overnight with eightfold water (w:v), decocted for two times (90 min per time), and then filtered. The filtrates were concentrated to 6–7 L by rotary vacuum evaporation and precipitated by adding 95% ethanol to a final concentration of 75%. After laying the mixture overnight, the supernatant was concentrated to 500 mL with a rotary evaporator. The final extract was stored in -20°C.

### Animals Treatment and Samples Collection

Male BALB/c mice (18–22 g, Silaike Co. Shanghai, China) were housed in controlled environmental conditions (12:12 h light/dark cycle and 25 ± 1°C) with conventional access to food and water. The mice were acclimatized for a period of 3 days prior to the start of the experiment. With the method of random grouping, mice were divided into two groups: the vehicle control group (*n* = 8, 1% sodium carboxymethyl cellulose) and the FMT water extract group (*n* = 16, 240 g/kg, an equivalent amount of the crude drug). Treatment was given at a frequency of intragastrical administration once a day for 3 days. In addition, individual body weight was recorded every day before oral gavage. Orbital blood samples were obtained after 6 h of the last oral gavage on the third day and centrifuged at 4°C, 4000 rpm for 15 min. The resulted serum was used for clinical biochemical analysis. 200 mg excised samples from the left lateral liver lobe were directly snap frozen in liquid nitrogen for microarray analysis (*n* = 5, chose the first five mice with higher ALT levels in FMT water extract treatment group, and randomly selected five mice in control group). The remaining liver lobes were used for histopathological examinations. All the protocols and studies involving the animals were conducted according to the Guiding Principles in the Use of Animals in Toxicology and the Animal Care and Use Committee of Zhejiang University School of Medicine.

### Biochemical Assay and Histopathological Examinations

The several parameters in serum were detected using Cobas C8000 system (Roche Diagnostics, Germany) according to the manufacturer’s instructions, including ALT, aspartate transaminase (AST), alkaline phosphatase (ALP), total bilirubin (TBILI), total cholesterol (TCHOL), and triglyceride (TG).

All livers were fixed with 10% formalin, embedded in paraffin, sectioned into 4-μm thick slices, mounted on poly(L-lysine)-treated slides, stained with hematoxylin and eosin, and examined by Olympus optical microscope.

### RNA Isolation

Total RNA, including miRNA, was extracted from frozen liver tissue using mirVana^TM^ miRNA Isolation Kit (Ambion, USA) following the manufacturer’s instructions and analyzed on an Agilent Bioanalyzer 2100 (Agilent technologies, USA). Only RNA Integrity Number (RIN) was greater than or equal to 7.0 and 28S/18S was greater than or equal to 0.7, the RNA sample was used for microarray analysis.

### miRNA Expression Analysis

The Agilent Mouse miRNA (8^∗^60 K) V19.0 array containing 1,247 miRNAs was used for miRNA analysis. After isolation, miRNA molecular in total RNA was labeled using miRNA Complete Labeling and Hyb Kit (Agilent technologies, USA) and hybridized in hybridization Oven (Agilent technologies, USA), following the manufacturer’s instructions. Raw data were transformed by log2 and then normalized by Quantile algorithm within Gene Spring GX 12.6.1 (Agilent technologies, USA). Data analysis was carried out using Welch *t*-test. A filter on low gene expression was used to keep only the probes expressed greater than 4 of the log2 transformed mean intensity in at least one sample. The miRNAs with *p*-value less than 0.05 and an absolute fold change more than 1.5-fold (Chang et al., 2011) between FMT water extract and vehicle control groups were considered to be significantly changed.

### mRNA Expression Analysis

mRNA expression profiles were obtained using the Affymetrix mouse 430 2.0 array as described in Lu et al. (2013). Raw data were imported into the ArrayTrack v 3.1.5 for further analysis. Microarray data were normalized by MAS 5.0 algorithm and were further normalized per chip to the same median intensity value of 1000. Welch *t*-test within ArrayTrack^®^ was used to identify the differentially expressed genes (DEGs) between FMT water extract and vehicle control groups with cutoffs of the *p*-value < 0.05, absolute fold change > 2, and the mean channel intensity more than 250.

### Real-time Quantitative PCR

To validate the results from microarray analysis, real-time quantitative PCR was carried out with the same RNA samples used for microarray analysis.

After reverse transcribed with miScript II RT Kit (Qiagen, Germany), three miRNAs were selected to validate the results from miRNAs microarray using real-time quantitative PCR with miScript SYBR Green PCR Kit (Qiagen, Germany) and individual specific primers (HuaDa, China) for miR-21a-3p (5′-CAACAGCAGTCGATGGGCTGTC-3′), miR-199a-5p (5′-CCCAGTGTTCAGACTACCTGTTC-3′), miR-139-5p (5′-TCTACAGTGCACGTGTCTCCAGT-3′), and U6 snRNA (5′- CTCGCTTCGGCAGCACA-3′). Furthermore, 11 mRNAs were chosen to validate the results from mRNAs microarray. After mRNAs were reverse transcribed with Oligo (dT)15 Primer (Promega, USA) and SuperScript II reverse transcriptase (Invitrogen, USA), real-time quantitative PCR was implemented for these testing mRNAs with QuantiFast SYBR Green PCR Kit (Qiagen, Germany) and individual specific primers from HuaDa (**Table 1**).

**Table 1:** Gene-specific primers used for real-time quantitative PCR amplification.

Gene Name	Forward primer	Reserve primer
*Car3*	5′-TGCTCAAAGAGCCCATGAC-3′	5′-GTGTTGTCGGACAGCTTGG-3′
*Tnfrsf12a*	5′-GCAGATCCTCGTGTTGGGA-3′	5′-CCACAGTAGCCTGAAGTGG-3′
*Cd14*	5′-TGATCTCAGCCCTCTGTCC-3′	5′- GGTACCTGCTTCAGCCCAG -3′
*Cidec*	5′-TACTTCCAAGCCCTGGCAA-3′	5′-CCTTCACGTTCAGGCAGCCAA-3′
*Cxcl2*	5′-GCTGTCAATGCCTGAAGACC-3′	5′-CTTCCGTTGAGGGACAGCA-3′
*Gadd45a*	5′-TGTGCTGGTGACGAACCCA-3′	5′-GTGCAGTTGAACTCGGCCCCT-3′
*S100a9*	5′-CTGACACCCTGAGCAAGAA-3′	5′-CTGTCACATGGCTGACCTC-3′
*Cyp4a14*	5′-GCCATTCTCAGGAGGATCAA-3′	5′-GTATTGCAGGCAGCAGACCTC-3′
*Map3k5*	5′-GTTTCTGGAACGTGGAGAGC-3′	5′-CTTCCCGAAAGCAGGGTC-3′
*Prkca*	5′-GTTTACCCGGCCAACGACT-3′	5′-GGGCGATGAATTTGTGGTCTT -3′
*Jun*	5′-CCTTCTACGACGATGCCCTC -3′	5′-GGTTCAAGGTCATGCTCTGTTT -3′
*GADPH*	5′-ACCAGGTTGTCTCCTGCGA-3′	5′-CAGTGTCCTTGCTGGGGTG-3′

All real-time quantitative PCR was performed on an Eppendorf Mastercycler ep realplex4 according to the manu facturer’s instructions. 2^-ΔΔCt^ method was used to generate the fold changes based on the previous study (Yang et al., 2011). Housekeeping gene U6 was a normalizer for these three miRNAs while GADPH was used to normalize the data for mRNAs.

### Ingenuity Pathways Analysis

The functions of differentially expressed miRNAs were further analyzed using Ingenuity Pathway Analysis software (IPA, Ingenuity Systems, USA). First, the target genes of differentially expressed miRNAs were predicted by IPA. Next, the intersection of the predicted target genes of differentially expressed miRNAs and the DEGs (*p*-value < 0.05, absolute fold change > 2) of FMT water extract treatment group was identified by IPA. The target mRNAs changed in the opposite direction of the corresponding miRNAs were selected for analysis by IPA Systems. Lastly, the biological and molecular functions of these genes were explored in IPA, such as relevant diseases and disorders, top canonical pathways, and top toxic lists. The canonical pathways of the DEGs were also performed by IPA as well as the network of the specific pathway.

### Statistical Analysis

Statistical analysis was performed by unpaired and two-tailed Student’s *t*-test. The level of significance was set at *p*-value < 0.05. Values are represented as mean ± standard deviation (SD) of triplicate determinations.

## Results

### Effects of FMT Water Extract on Mouse Body Weight and Liver Histopathology

**FIGURE 2:**
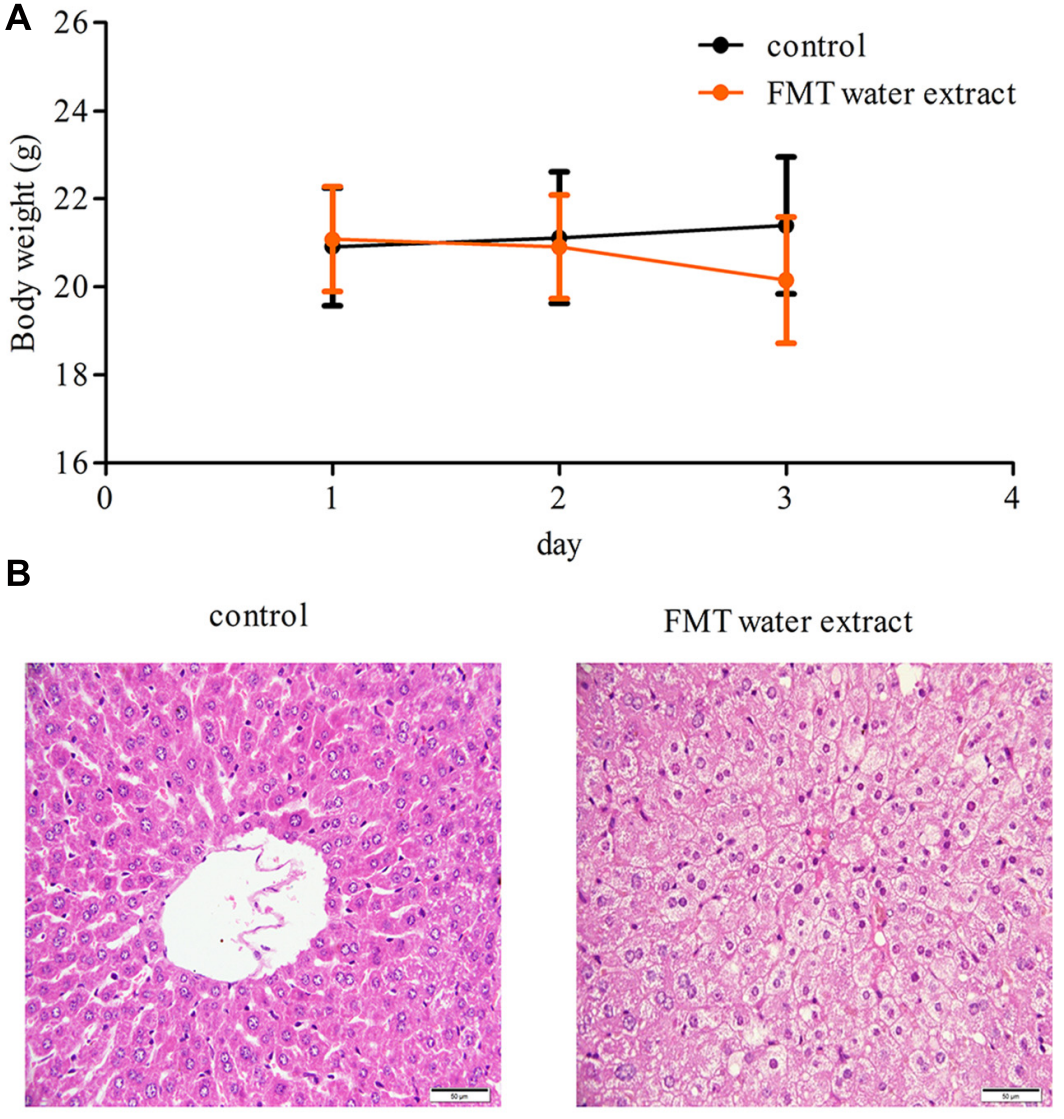
**(A)** The body weights of mice treated with Fructus Meliae Toosendan (FMT) water extract. The results showed that there were no significant changes in body weight between vehicle control (1% sodium carboxymethyl cellulose, *n* = 8) and FMT water extract groups (240 g/kg, *n* = 16) by oral gavage for 3 days; **(B)** Histological analysis of the liver tissues of FMT water extract-treated mice. Hydropic degeneration of hepatocytes was detected in FMT water extract-treated mice. The scale bar is 50 μm.

Compared with control group, there were no significant changes observed in body weight in FMT water extract group at any time point (**Figure 2A**). Two mice were dead in FMT water extract group at the third day after exposure to FMT water extract. Moreover, hydropic degeneration of hepatocytes was detected in the livers of FMT water extract-treated mice, whereas the livers of vehicle control group exhibited normal histology in histopathological examinations (**Figure 2B**).

### Effects of FMT Water Extract on Serum Biochemistry

**FIGURE 3:**
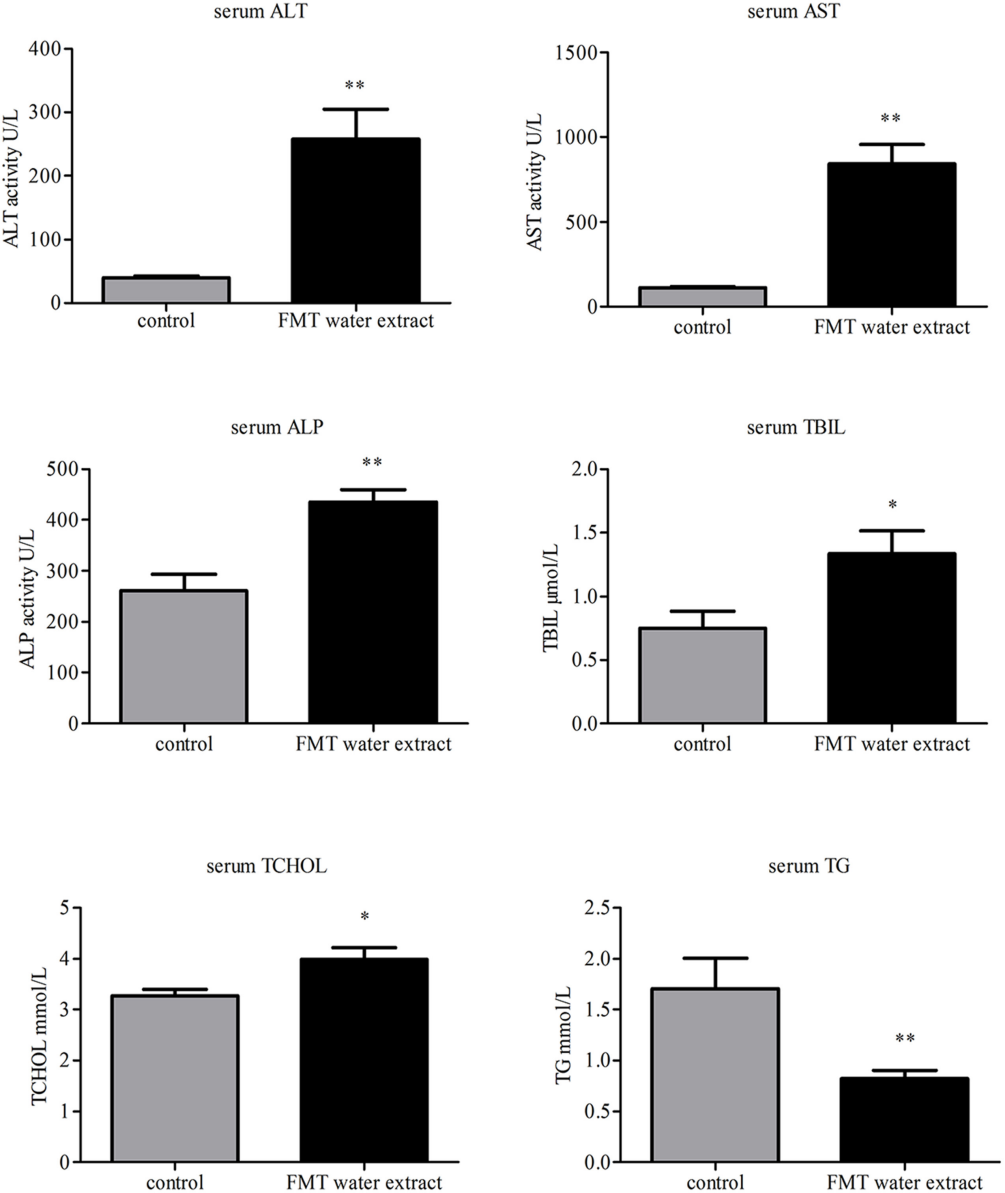
**Effects of FMT water extract on serum biochemical parameters after intragastric administration of 240 g/kg FMT water extract for 3 days in mice (control group: *n* = 8; FMT water extract group: *n* = 14).** Values are represented as mean ± SD (^∗^*p* < 0.05, ^∗∗^*p* < 0.01 compared with vehicle control group).

The biochemical parameters, including ALT, AST, ALP, TG, TBIL, and TCHOL, were used as the biochemical markers to monitor FMT water extract-induced liver injury. As shown in **Figure 3**, intragastric administration of FMT water extract (240 g/kg) caused significant increases in serum TBIL and TCHOL levels as well as ALT, AST, and ALP activities compared with control group, whereas remarkable decrease of TG level in FMT water extract group was detected.

### Effects of FMT Water Extract on miRNA Expression Profiles

**FIGURE 4:**
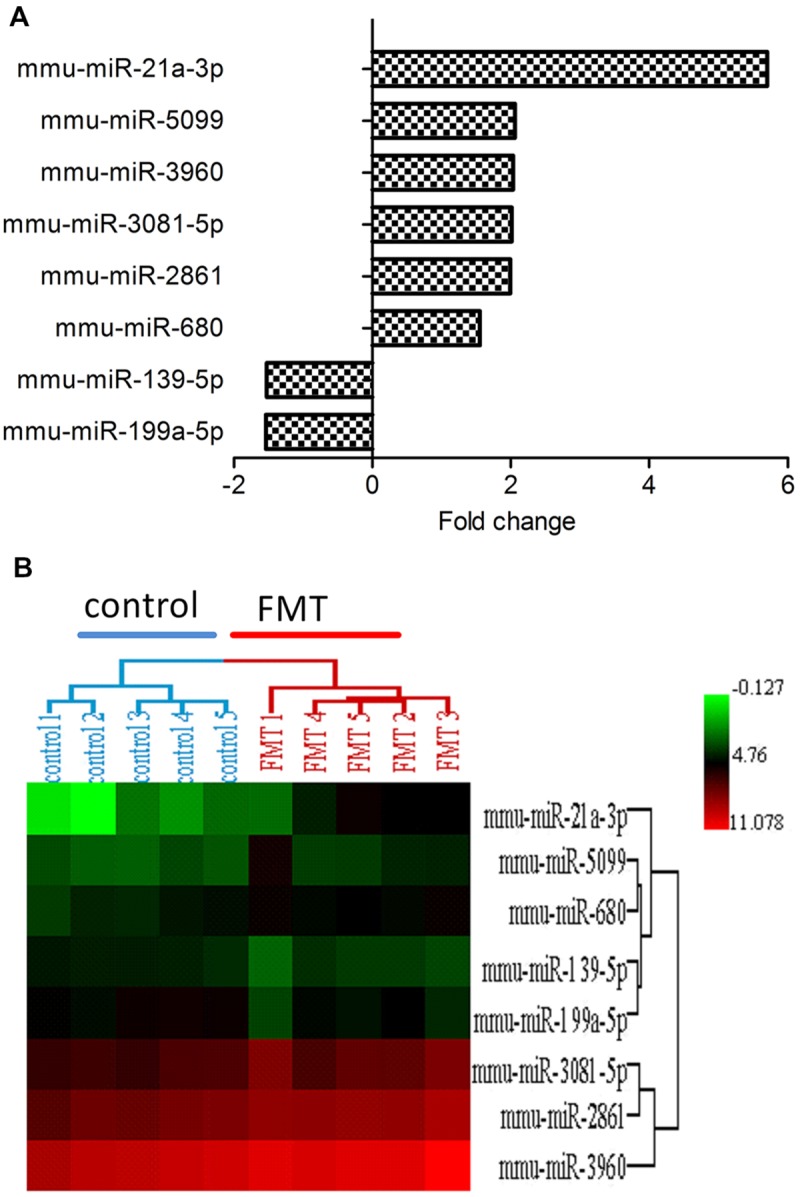
**(**A**)** The differentially expressed miRNAs in FMT water extract group. There were eight miRNAs (0.64%) of total 1,247 studied miRNAs differentially expressed in FMT water extract group when compared with control group (*n* = 5). six miRNAs were upregulated (fold change > 1.5) and 2 miRNAs were downregulated (fold change < -1.5); **(B)** Hierarchical Cluster Analysis (HCA) within ArrayTrack^®^ was used to visualize clusters of samples corresponding to the differentially expressed miRNAs. There were two main branches detected (FMT water extract-treated mice versus control mice).

**FIGURE 5:**
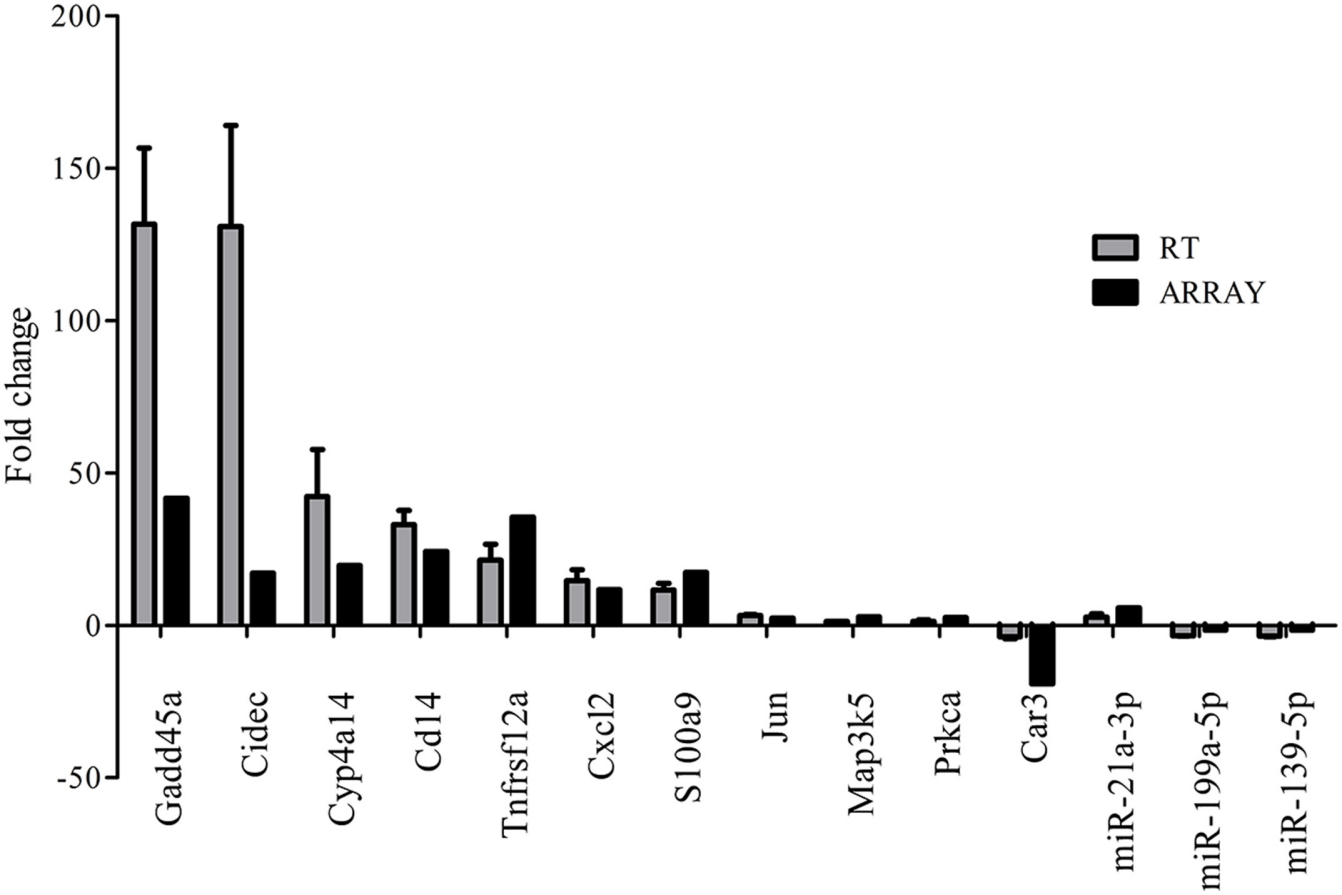
**Validation of microarray results by real-time quantitative PCR.** Gray bars (RT) indicate the mean fold change (±SD) derived from triplicate real-time quantitative PCR reactions (*n* = 5). Black bars (ARRAY) indicate microarray data.

miRNA expression profiles were analyzed with Agilent Mouse miRNA (8^∗^60 K) V19.0 array. Eight miRNAs (approximately 0.64%) of total 1,247 miRNAs investigated were differentially expressed in FMT water extract-treated group (**Figure 4A**). Among these miRNAs, six miRNAs were upregulated over 1.5-fold, whereas two miRNAs were downregulated over 1.5-fold. Hierarchical cluster analysis (HCA) within ArrayTrack^®^ was used to visualize clusters of the liver samples corresponding to the differentially expressed miRNAs (**Figure 4B**). The results showed that all samples were grouped into two main branches (FMT water extract-treated mice versus control mice). Three of these eight miRNAs were verified by real-time quantitative PCR. It was demonstrated that the expression of miR-21a-3p was upregulated and miR-199a-5p and miR-139-5p were downregulated in FMT water extract-treated group when compared with control group (**Figure 5**), which was consistent with the results from microarray analysis.

### Effects of FMT Water Extract on mRNA Expression Profiles

**FIGURE 6:**
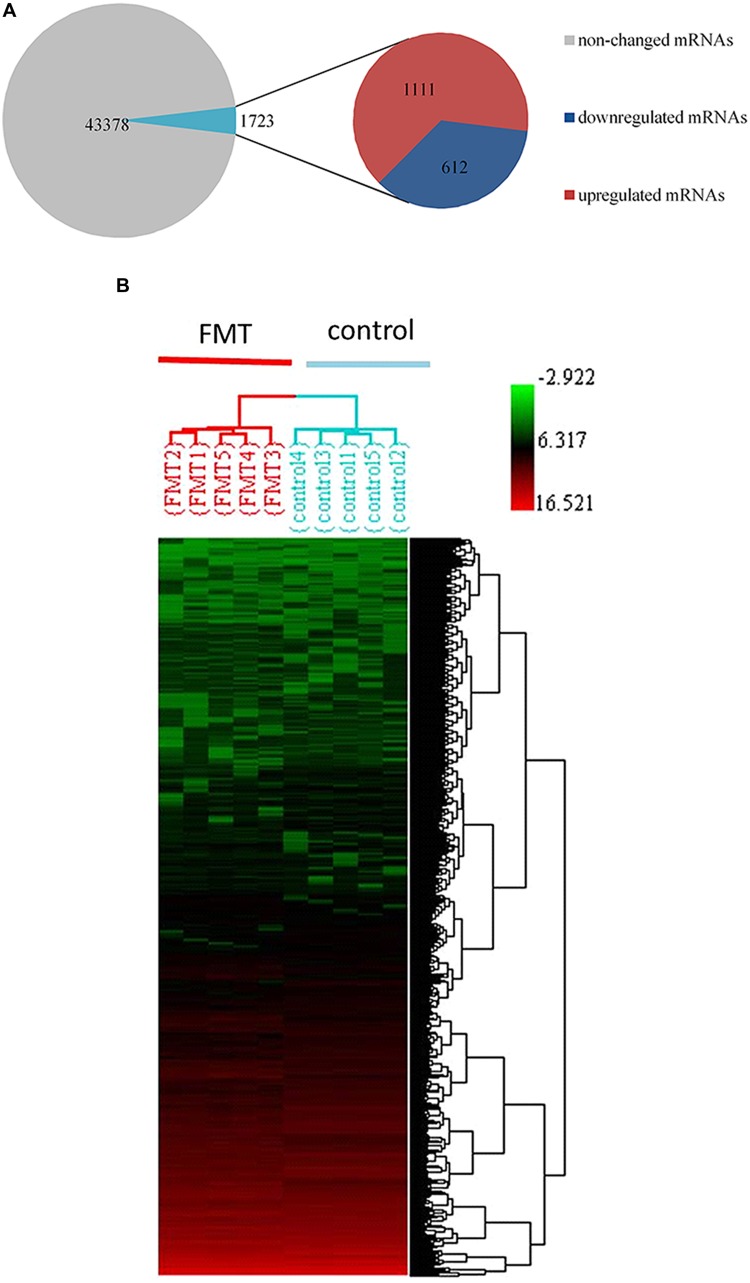
**(A)** 1723 mRNAs (3.8%) of total 45,101 genes were significantly changed by FMT water extract administration detected by Affymetrix mouse 430 2.0 array. Compared with control group, 1,111mRNAs were upregulated (fold change > 2) as well as 612 mRNAs downregulated (fold change < -2); **(B)** HCA within ArrayTrack was used to visualize clusters of samples corresponding to the DEGs. There were two main branches detected (FMT water extract-treated mice versus control mice).

We observed that approximately 3.8% (1723 mRNAs) of total genes (45,101 mRNAs) were significantly changed in the liver of FMT water extract-treated mice (**Figure 6A**). Among them, 1,111 mRNAs were upregulated more than twofold (*p* < 0.05) and 612 mRNAs were downregulated more than twofold (*p* < 0.05) in FMT water extract group compared with control group. Moreover, HCA results displayed that all samples were grouped into two main branches (FMT water extract-treated mice versus control mice, **Figure 6B**). To validate the results obtained from mRNA microarray, the expression levels of 11 DEGs were detected by real-time quantitative PCR. As a result, the general trends observed in the microarray analysis of all genes tested *(Gadd45a, Cidec, Cyp4a14, Cd14, Tnfrsf12a, Cxcl2, S100a9, Jun, Map3k5, Prkca, and Car3)* were verified by real-time quantitative PCR (**Figure 5**).

### Prediction of Biological Function and Disease Establishment Associated with Intersection of miRNAs Targets and DEGs

Based on Affymetrix probe ID, 1594 of 1723 DEGs in FMT water extract treatment could be mapped in IPA. Moreover, the target genes of ***four*** miRNAs could be predicted by target filter analysis in IPA database, including miR-139-5p, miR-199a-5p, miR-2861, and miR-3960. As miRNAs usually decrease the expressions of target mRNAs, the intersection of the 1594 DEGs and the predicted target genes of these ***four*** miRNAs was identified, which was based on the opposite expression direction between miRNAs and mRNAs. As the results, 125 mRNAs showed an opposite expression trend compared with corresponding miRNA. Furthermore, the intersected 125 mRNAs were analyzed to predict biological functions using IPA bioinformatics tool. We found that most of the genes were involved in molecular and cellular functions, such as cellular development, growth and proliferation, gene expression, cell morphology, and cellular assembly and organization (**Table 2**). In addition, the top 5 canonical pathways (according to the *p-*value for these 125 mRNAs were involved in tight junction signaling, retinoid acid receptor (RAR) activation, NF-E2-related factor 2 (NRF2)-mediated oxidative stress response, hepatocyte growth factor (HGF) signaling, and planar cell polarity (PCP) pathway (**Table 3**). In top 5 toxic lists (according to the *p-*value), liver necrosis or cell death was the most relevant toxicity for FMT water extract-induced liver injury (**Table 4**).

**Table 2:** Top five cellular functions predicted by Ingenuity Pathway Analysis (IPA) that were corresponding to 125 mRNAs of the intersection of the four differentially expressed miRNAs predicted target genes and differentially expressed genes (DEGs) under Fructus Meliae Toosendan (FMT) water extract treatment.

Molecular and cellular functions	Number of genes involved	*p-*value
Cellular development	46	2.60E-07–1.15E-02
Cellular growth and proliferation	44	2.60E-07–1.15E-02
Gene expression	36	3.13E-06–1.15E-02
Cell morphology	38	4.64E-06–1.15E-02
Cellular assembly and organization	31	4.64E-06–1.15E-02

**Table 3:** Top five canonical pathways predicted by IPA that were corresponding to 125 mRNAs of the intersection of the four differentially expressed miRNAs predicted target genes and DEGs under FMT water extract treatment.

Canonical pathways	-log(*p*-value)	Molecules
Tight junction signaling	3.38	*CPSF2, JUN, MYH9, PPP2CA, NAPG, GOSR1*
Retinoid acid receptor (RAR) activation	3.26	*TRIM24, JUN, SMARCD2, MAP3K5, PML, PRKCA*
NRF2-mediated oxidative stress response	3.21	*JUN, STIP1, JUNB, MAP3K5, ENC1, PRKCA*
Hepatocyte growth factor (HGF) signaling	2.49	*JUN, ETS2, MAP3K5, PRKCA*
Planar cell polarity (PCP) pathway	2.24	*JUN, FZD6, JUNB*

**Table 4:** Top five toxic lists predicted by IPA that were corresponding to 125 mRNAs of the intersection of the four differentially expressed miRNAs predicted target genes and DEGs under FMT water extract treatment.

Tox lists	-log(*p*-value)	Molecules
Liver necrosis/Cell death	3.73	*SPTBN1, MNT, ARF6, JUN, ABL2, IGFBP1, SERPINE1, MCL1*
Cardiac hypertrophy	3.43	*MNT, EPAS1, JUN, ATF3, HIF1A, MAP3K5, SERPINE1, PRKCA, DYRK1A*
NRF2-mediated oxidative stress response	3.38	*JUN, CYP2U1, STIP1, JUNB, MAP3K5, ENC1, PRKCA*
Acute renal failure panel	3.34	*LIFR, JUN, ATF3, IGFBP1*
RAR activation	3.26	*TRIM24, JUN, SMARCD2, MAP3K5, PML, PRKCA*

### The Network of NRF2-mediated Oxidative Stress Response Associated with DEGs and Differentially Expressed miRNAs

**FIGURE 7:**
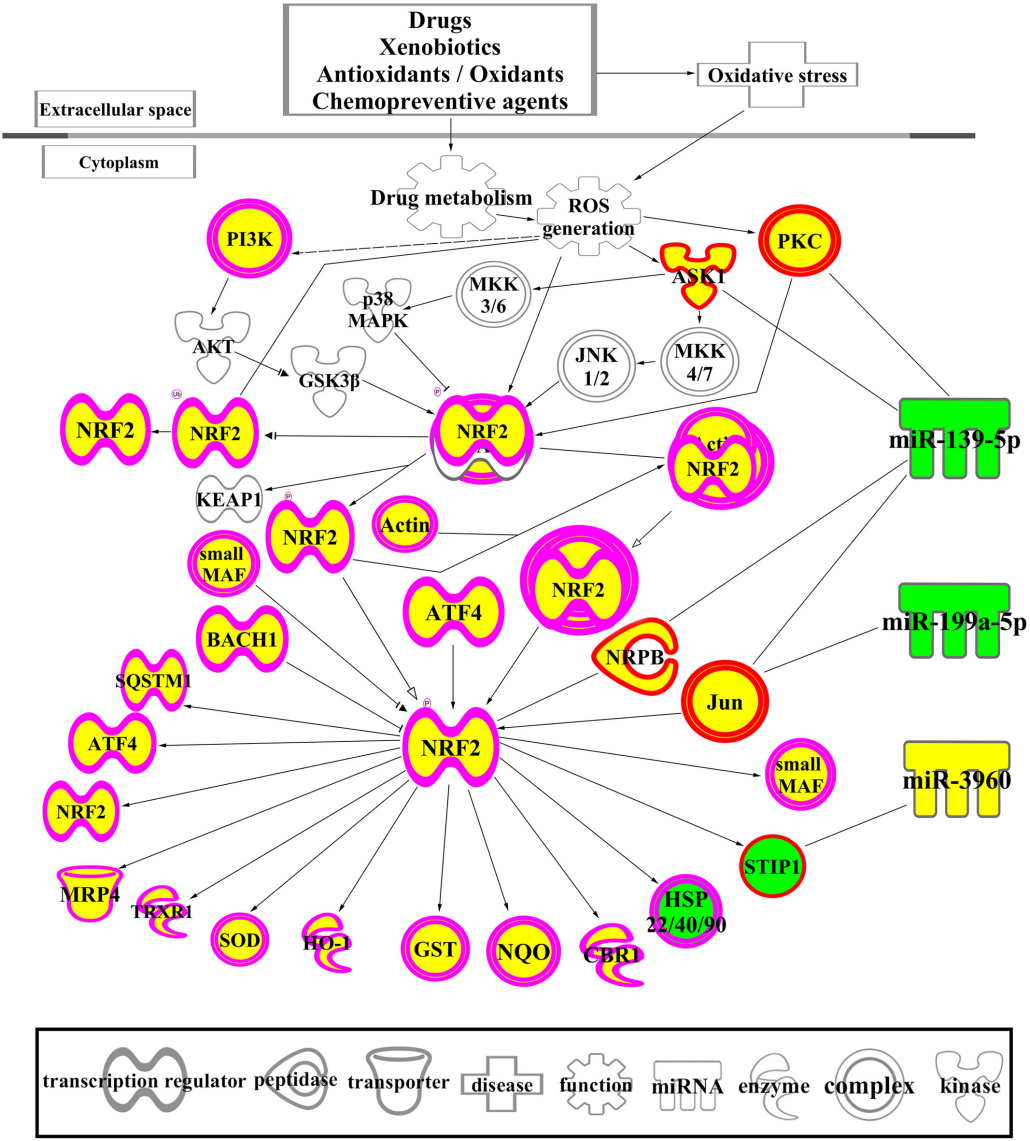
**A network of NRF2-mediated oxidative stress response associated with DEGs and differentially expressed miRNAs.** The yellow color means the up-regulation of the mRNAs and miRNAs after exposure to FMT water extract compared with control group, while the green color indicates the down-regulation of these molecules after exposure to FMT water extract. Different shapes outside of the molecules are illustrated at the figure.

The biological functions of the 1594 DEGs in FMT water extract treatment were analyzed by IPA. As shown in **Table 5**, the NRF2-mediated oxidative stress response was the most significant canonical pathway enriched in this dataset, which was also predicted by the 125 intersected mRNAs (**Table 3**). With the combination of the affected miRNAs and mRNAs, a network of NRF2-mediated oxidative stress response was generated by IPA and shown in **Figure 7**. As the results, lots of signaling molecules in this pathway were interfered by FMT water extract treatment.

**Table 5:** Top five canonical pathways predicted by IPA that were corresponding to the 1594 DEGs of FMT water extract treatment.

Canonical pathways	-log(*p*-value)	Number of associated molecules
NRF2-mediated oxidative stress response	8.96	32
Glucocorticoid receptor signaling	6.41	36
Unfolded protein response	6.31	14
P38 MAPK signaling	4.99	19
Activation of IRF by cytosolic pattern recognition receptors	4.65	13

## Discussion

Traditional Chinese Medicine has been widely employed around the world in many diseases, however, liver injury is one of the main side effects after exposure to TCM and it has been increased in recent years (Wai, 2006). As the components of TCM are very plentiful, it is complicated to explore the mechanism of TCM-induced liver injury. Shedding some light on this issue, miRNA microarray has already provided insights into the mechanisms and biomarkers of chemical DILI (Fukushima et al., 2007), which could be a new approach to study TCM-induced liver injury. Thus, in this study, we first applied an integrated approach using mRNA and miRNA microarray to study the molecular mechanisms of TCM-induced liver injury and chose FMT as a model drug.

Serum aminotransferases are the standard biomarkers for liver injury. When liver injured, the activities of ALP, ALT, and AST in serum could increase. Studies reported that the serum levels of ALT and AST were correlated with the degree of the liver injury (Myers et al., 2003; Lee et al., 2004). In this study, traditional serum transaminases (AST, ALT, and ALP) from FMT water extract group were higher than the control group (*p* < 0.01), indicating liver injury induced by FMT water extract treatment in mice. It may be caused by cell necrosis as the increases of cell membrane permeability which allowed the transaminases leaking from the cells. Consistent with that, hydropic degeneration of hepatocytes was detected in FMT water extract-treated mice. When hepatocytes were swelling, it might decrease albumin production and result in increasing bilirubin in the blood at the later stage (Lu et al., 2011). Concordantly, an increased level of serum TBIL was observed by FMT water extract treatment. The decreased level of TG was also detected after FMT water extract treatment, indicating the impact of FMT water extract on lipid metabolism. Thus, changes in serum biochemistry and histopathology indicated that FMT water extract could cause liver injury.

There were eight miRNAs differentially expressed in livers of FMT water extract-treated mice (**Figure 4A**). Interestingly, miR-122 and miR-192 did not show any differences between these two groups in our experiment though they are the biomarkers for liver injury (Su et al., 2012). Similarly, Bala et al. (2012) reported that hepatic miR-122 did not change while circulating miR-122 increased in DILI. The possible explanation was that the distinct experiment samples could lead to the contrary results. In our experiment, we chose livers as the source of miRNAs while these two biomarkers (miR-122 and miR-192) were proved to be useful in circulating miRNA (Starkey et al., 2011). The circulating miRNAs are transported by exosomes, microparticles, lipoproteins, and other ribonucleoprotein complexes (Kosaka et al., 2010; Arroyo et al., 2011; Boon and Vickers, 2013). It demonstrated that the miRNA profiles of extracellular vesicles and lipoproteins are not representative of their parent cell type but are distinct sets of miRNAs (Valadi et al., 2007; Wang et al., 2010; Zhang et al., 2010), and probably reach many distant tissues to modulate systemic homeostasis or promote the disease (Boon and Vickers, 2013). In this study, the cardiac hypertrophy and acute renal failure panel were also predicted in the top 5 toxic lists by IPA, indicating these distant organs might be also affected by FMT water extract or the secondary responses of extracellular miRNAs in liver toxicity. It partly corresponded with the reports that FMT can induce hepatotoxicity, cardiotoxicity, and nephrotoxicity (Lihua et al., 2006; Shuangyan et al., 2008).

Although only hydropic degeneration of hepatocytes rather than hepatocytes necrosis was detected in the livers of FMT water extract-treated mice by histopathological examinations, the toxic lists in IPA showed that the main toxicity of FMT water extract was liver necrosis or cell death. The possible reason for the phenomenon is that the alterations in gene expression are always earlier than the changes in protein and histopathological levels (Beer et al., 2002). In this study, we focused on liver necrosis or cell death as it was the most significant pathway enriched in toxic lists and it corresponded to the other canonical pathways predicted by IPA after exposure to FMT water extract (**Tables 3** and **4**), such as HGF signaling, RAR activation, NRF2-mediated oxidative stress response, tight junction signaling, and PCP pathway. More specifically, HGF was a multifunctional cytokine that stimulated cell necrosis (Suzuki et al., 2000). Wang et al. (2007) reported that activation of RAR alpha (retinoic acid receptors) could inhibit Nrf2, which regulated detoxification processes (Chan et al., 2001). NRF2-mediated oxidative stress related to liver diseases (Cederbaum et al., 2009) and participated in cell death, proliferation and differentiation (Richter and Kass, 1991). In addition, tight junction, which was detected in flutamide-induced liver toxicity (Legendre et al., 2014), played an important role in cell apoptosis (Zhang et al., 2011). PCP pathway was a non-canonical Wnt signaling pathway, which directed processes such as cell fate determination, proliferation, and differentiation (Teoa and Kahn, 2010). Thus, it indicated that these pathways related to cell necrosis or death and may play important roles in FMT water extract-induced liver injury.

Corresponding to the canonical pathways revealed by the 125 intersected mRNAs, the NRF2-mediated oxidative stress response was also enriched as the most significant canonical pathway by the 1594 DEGs of FMT water extract group. Thus, we speculated that the oxidative stress might be the primary cause of FMT-induced liver damage. As shown in **Figure 7**, lots of signaling molecules in the NRF2-mediated oxidative stress response were interfered by FMT water extract treatment. We noticed that ASK1, PKC, and JUN affected by the differentially expressed miRNAs of FMT water extract, i.e., miR-139-5p and miR-199-5p. The formers were the upstream molecules of NRF2 and played important roles in oxidative stress response. In particular, ASK1, known as Map3k5, was the target of miR-139-5p. It mediated signal transduction of various stressors such as oxidative stress (Noguchi et al., 2005), and played a crucial role in cell survival (Zhang et al., 1999) and hepatic proliferation (Noguchi et al., 2014). PRKCA, a member of PRC, was the target of miR-139-5p. It was activated in oxidative stress-related diseases, such as cancer, cerebral ischemia-reperfusion injury, hepatic damage, and involved in positive and negative regulation of cell proliferation, apoptosis, differentiation, and cell injury (Mandil et al., 2001; Nitti et al., 2008; Kamo et al., 2011). JUN was the common molecule of NRF2-mediated oxidative stress response and cell necrosis from toxic lists of the 125 intersected mRNAs. It was regulated by miR-139-5p and miR-199a-5p and activated by the oxidative stress (Lee et al., 2001). Previous studies reported that JUN worked as a regulator of cell fate, such as hepatocyte survival, proliferation, and liver tumorigenesis (Kayahara et al., 2005; Fuest et al., 2012). Taken together, oxidative stress and subsequent liver cell necrosis may be the toxicological mechanisms of FMT-induced liver injury.

A literature-based analysis was performed to increase evidence of the IPA results for the four miRNAs with targets in our experimental data. These miRNAs were involved in some processes such as cellular growth and proliferation, gene expression, cancer, and cellular development. For example, miR-3960 regulated cellular growth and proliferation through a regulatory feedback loop with miR-2861 (Hu et al., 2011; Wong et al., 2011), meanwhile, these two miRNAs could relate with oxidative stress response (Dalle et al., 2012). The deregulation of miR-2861 and miR-21a-3p was also related to cancer diseases (Wang et al., 2013; Gordillo et al., 2014). In addition, miR-139-5p was a tumor-suppressor miRNA and had been found downregulated in hepatoma cells samples when compared with adjacent benign tissues (Jiang et al., 2008; Au et al., 2012; Huang et al., 2012). Similarly, miR-199a-5p, one of the most abundant miRNAs in hepatocytes, prevented hepatocyte apoptosis (Dai et al., 2013). Thus, the downregulation of miR-139-5p and miR-199a-5p in this study may indicate the potential risk of hepatic carcinoma caused by FMT water extract. Moreover, the dysregulation of miR-21a and miR-199a-5p have been also reported by other liver injury models. Specifically, the increase of miR-21a expression was detected in alcoholic liver injury (Francis et al., 2014), and the expression of miR-199a-5p was elevated in both bile acid- and thapsigargin-stimulated cultured hepatocytes, as well as in the liver of bile duct-ligated mice (Dai et al., 2013). In addition, since the microarray results might not come exclusively from hepatocytes as the liver tissue used for this analysis, we further discussed the possible contribution of the other cell types in liver to the pathology and the expression profile of miRNAs. Among these four miRNAs, the miR-199a-5p and miRNA-21 were found to be expressed by other cell types of liver and contributed to liver diseases. For instance, hepatic stellate cells had been found to express miR-199, which influenced stellate cell activity by regulating collagen synthesis and represented a general mechanism contributing to hepatic fibrosis (Lino et al., 2013); the liver sinusoidal endothelial cells also express the miR-199 and this miRNA might serve as a negative regulator of the function of liver sinusoidal endothelial cells in some chemical exposures, such as alcohol (Wang et al., 2012); the increased miR-21 expression was detected in the hepatic stellate cells with the treatment of ethanol and interleukin-6 (Francis et al., 2014).

## Conclusion

Fructus Meliae Toosendan water extract can induce the liver injury in a mice model, and integrated microRNA-mRNA approach provided insights into the molecular mechanisms of FMT water extract-induced liver injury, which demonstrated the feasibility of applying a microRNA-mRNA network based approach to study TCM-induced liver injury.

## Conflict of Interest Statement

The authors declare that the research was conducted in the absence of any commercial or financial relationships that could be construed as a potential conflict of interest.
